# 668. Restricting Ordering of Multiplex Gastrointestinal Panel Improves Test Utilization

**DOI:** 10.1093/ofid/ofab466.865

**Published:** 2021-12-04

**Authors:** Don Bambino Geno Tai, Anisha Misra, Priya Sampathkumar, John C O'Horo

**Affiliations:** Mayo Clinic, Rochester, Minnesota

## Abstract

**Background:**

The multiplex gastrointestinal pathogen panel (GIP) is a convenient and quick diagnostic test for determining the infectious etiology of diarrhea. It identifies several of the most common pathogens associated with gastroenteritis. However, it is expensive, and test results may not impact care, given that several of the pathogens in the panel are managed expectantly. We describe our experience with a diagnostic stewardship initiative to resolve the overuse of this testing method.

**Methods:**

We performed a pre/post study of GIPs ordered for inpatients 18 years old and older from December 19, 2018, to December 18, 2020, at Mayo Clinic hospital in Rochester, Minnesota. GIP orders for inpatients were limited to the first 72 hours of hospitalization starting December 19, 2019. Orders after 72 hours were encouraged to be changed to *Clostridioides difficile* NAAT testing or sent to an infectious disease provider to override on a case-by-case basis. Our hospitals used BioFire® FilmArray® Gastrointestinal Panel (BioFire Diagnostics, Salt Lake City, Utah).

**Results:**

A total of 2,641 GIPs were performed during the study period. There were 1,568 GIPs (3.3/100 hospitalizations) in the pre-intervention period compared to 1,073 (2.6/100 hospitalizations) post-intervention, representing a drop of 21.2%. The most common pathogen detected was *C. difficile* (toxin A/B) (48.8%, n=402), followed by norovirus (17.5%, n=144). The overall test positivity rate was 27.9% (n=736). The test positivity rate decreased 1.8% from 28.6% (n=448) to 26.8% (n=288) after the restriction (p=0.33). The proportion of *C. difficile* among all pathogens detected increased from 48.5% to 49.7% (*p*=0.67).

Table 1. Pre- and Post-Intervention Test Positivity Rate of Specific Pathogens in GIP

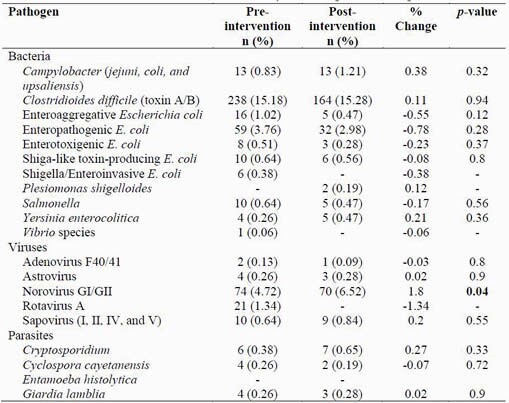

**Conclusion:**

Our study showed that restricting the ordering of GIP to the first 72 hours of hospitalization and directing providers to standalone *C. difficile* NAAT testing resulted in a reduction of GIPs performed. There were marginal changes in the test positivity rate of GIP. A limitation of our study is that the timing of post-intervention coincided with the COVID-19 pandemic, which had unpredictable effects on hospital practice and patient admissions. Ideally, future quality improvement projects should increase the test positivity of pathogens other than *C. difficile* while lowering the GIP use in diagnosing *C. difficile* colitis.

**Disclosures:**

**John C. O'Horo, Sr., MD, MPH**, **Bates College and Elsevier Inc** (Consultant)

